# Mid-Gestation lethality of *Atxn2l*-Ablated Mice

**DOI:** 10.3390/ijms21145124

**Published:** 2020-07-20

**Authors:** Jana Key, Patrick N. Harter, Nesli-Ece Sen, Elise Gradhand, Georg Auburger, Suzana Gispert

**Affiliations:** 1 Exp. Neurology, Medical Faculty, Goethe University, Theodor Stern Kai 7, 60590 Frankfurt am Main, Germany; key@stud.uni-frankfurt.de (J.K.); nesliecesen@gmail.com (N.-E.S.); 2 Faculty of Biosciences, Goethe-University, Altenhöferallee 1, 60438 Frankfurt am Main, Germany; 3 Institute of Neurology (Edinger-Institute), University Hospital Frankfurt, Goethe University, Heinrich-Hoffmann-Strasse 7, 60528 Frankfurt am Main, Germany; patrick.harter@kgu.de; 4 Dr. Senckenberg Institute for Pathology, University Hospital, Goethe University, Theodor-Stern-Kai-7, 60590 Frankfurt am Main, Germany; elise.gradhand@kgu.de

**Keywords:** poly(A)-tail, RNA chaperone, Spinocerebellar ataxia type 2, SCA2, tauopathy, fronto-temporal lobar dementia, tyrosine kinase receptor signaling, nutrient endocytosis

## Abstract

Depletion of yeast/fly Ataxin-2 rescues TDP-43 overexpression toxicity. In mouse models of Amyotrophic Lateral Sclerosis via TDP-43 overexpression, depletion of its ortholog ATXN2 mitigated motor neuron degeneration and extended lifespan from 25 days to >300 days. There is another ortholog in mammals, named ATXN2L (Ataxin-2-like), which is almost uncharacterized but also functions in RNA surveillance at stress granules. We generated mice with Crispr/Cas9-mediated deletion of *Atxn2l* exons 5-8, studying homozygotes prenatally and heterozygotes during aging. Our novel findings indicate that ATXN2L absence triggers mid-gestational embryonic lethality, affecting female animals more strongly. Weight and development stages of homozygous mutants were reduced. Placenta phenotypes were not apparent, but brain histology showed lamination defects and apoptosis. Aged heterozygotes showed no locomotor deficits or weight loss over 12 months. Null mutants in vivo displayed compensatory efforts to maximize *Atxn2l* expression, which were prevented upon nutrient abundance in vitro. Mouse embryonal fibroblast cells revealed more multinucleated giant cells upon ATXN2L deficiency. In addition, in human neural cells, transcript levels of *ATXN2L* were induced upon starvation and glucose and amino acids exposure, but this induction was partially prevented by serum or low cholesterol administration. Neither ATXN2L depletion triggered dysregulation of ATXN2, nor a converse effect was observed. Overall, this essential role of ATXN2L for embryogenesis raises questions about its role in neurodegenerative diseases and neuroprotective therapies.

## 1. Introduction

In all eukaryotic organisms, at least one copy of the Ataxin-2 gene (gene symbol *ATXN2* in humans) is phylogenetically conserved and serves roles during nutrient stress for RNA surveillance [1]. A conserved Lsm and LsmAD motif enables direct interaction with RNAs, and a PAM2 motif mediates association with the poly(A)-binding protein PABPC1 [2,3]. Thus, most Ataxin-2 protein localizes with mRNAs at the rough endoplasmic reticulum with marker Ribosomal Protein S6 (RPS6 aka S6R) during cell growth periods [4], where its absence leads to expression adaptations of the associated ribosomal translation machinery [5] and modulates the phosphorylation control of translation [6]. During cell stress, e.g., from nutrient deprivation, Ataxin-2 is transcriptionally induced [6] and relocates with the small ribosomal subunit and PABPC1 to stress granules (SG) [7]. The RNA surveillance function of Ataxin-2 seems to be relevant to protect against the translation of viral RNAs, given that poliovirus is optimized to cleave Ataxin-2 [8]. In mammals, all these protein structure domains are also found in its paralog Ataxin-2-like (gene symbol *ATXN2L* in humans). Both *ATXN2* and *ATXN2L* mRNAs also conserved an alternatively spliced exon, which encodes a proline-rich domain (PRD) that mediates its direct association with SH3 motifs in components of the growth factor receptor endocytosis apparatus [9,10,11]. Abnormal *ATXN2* splicing and alternative polyadenylation were documented in diseases with RNA toxicity, such as amyotrophic lateral sclerosis (ALS) [12]. The common ancestor of both proteins in yeast and *Caenorhabditis elegans* was observed to suppress growth signaling via mTORC1, modulating cell size, and lipid stores [13,14,15]. This reprogramming of nutrient metabolism is accompanied by an important influence on the mitochondrial breakdown of fatty acids and amino acids, as well as glucose utilization [16,17,18,19], probably mediated by the direct protein interaction of ATXN2 with the cytosolic enzyme BCAT1 [20] as the rate-limiting factor in the breakdown of leucine, isoleucine, and valine. 

The role of ATXN2 in neurodegenerative diseases has triggered intense research over the past 25 years. Exclusively in human ATXN2, an N-terminal domain with 22 consecutive glutamines (polyQ) exists, which can undergo expansion mutations across generations. Large expansions beyond the size of 32Q trigger the multi-system nervous tissue atrophy Spinocerebellar ataxia type 2 (SCA2), while intermediate expansions of sizes 27Q-32Q increase the risk to be affected by motor neuron diseases such as ALS, fronto-temporal lobar dementia (FTLD) [21,22,23,24,25] or by other tauopathies and Parkinson’s disease variants like progressive supranuclear palsy (PSP) [26,27]. Conversely, the depletion of ATXN2 by knock-out or by injection of antisense-oligonucleotides has a massive neuroprotective effect in yeast/fly/mouse models of ALS and FTLD, as well as in SCA2 and SCA1 fly models [24,27,28,29]. In addition, in yeast, depletion of the ATXN2/ATXN2L ortholog PBP1 rescues the lethal effect of poly(A)-binding protein deletions [30]. The constitutive knock-out of *Atxn2* in mice leads to progressive weight gain with excessive storage of lipid droplets and glycogen in the liver, elevated cholesterol and other lipids in the blood, beta-cell hyperplasia in the pancreas with hyperinsulinemia and insulin resistance, increased ganglioside and sulfatide lipids in the brain myelin, locomotor hyperactivity, and mild infertility with gender-dependent impairment of embryogenesis [31,32].

In view of the importance of ATXN2 orthologs for stress response, redundancy occurred in land plants and in vertebrates (except birds) by the co-existence of two homologous genes, named *CID3*-*CID4* in *Arabidopsis thaliana* weed and *ATXN2*-*ATXN2L* in humans and rodents [33]. ATXN2L protein dimerizes with ATXN2 in yeast-two-hybrid tests, and is also a regulator of SGs and mRNA processing during starvation periods, but shows more co-localization with the nuclear splice apparatus than ATXN2 due to an arginine-dimethylation [34,35]. Similar to ATXN2, ATXN2L associates with plasma membrane receptors in dependence on their phosphorylation status, is involved in epidermal-growth-factor (EGF)-receptor signaling, and exists in several isoforms [36,37,38]. Little more is known at present about ATXN2L. Database mining at the STRING web platform for Protein-Protein Interaction Networks and Functional Enrichment Analysis, available online at: https://string-db.org/ [39] confirms that human and mouse ATXN2L show direct protein-protein-interaction with the poly(A)-binding-proteins PABPC1/2, PABPC1L/PABPC3/PABPC4/PABPC6, with further stress-granule-components such as G3BP2, DDX6, and LSM12 [40,41], as well as nuclear RNA-binding protein NUFIP2 and nuclear transcription modulator NFATC2IP. The RNA helicase activity of DDX6 enhances the expression of growth factor receptors such as HER2 and FGFR2 [42]. Possibly by this mechanism, ATXN2 was found to modulate surface receptors [43]. A recent global protein interactome study via FLAG-tag affinity purification and mass spectrometry in HEK293 cells also showed ATXN2L to associate with DDX6 and the proline-rich-domain-containing RNA-binding Ras-GTPase G3BP2, but in addition, demonstrated ATXN2L binding to KLHL20 [20], which is responsible for the degradation of the autophagy/growth modulators DAPK1 and ULK1, as well as of the Rho Guanine nucleotide exchange factor and glutamate-transport-/neurite-growth-modulator ARHGEF11. STRING also lists a potential interaction (based on association in lower species) with SLC9A3R2/NHERF2 that is known to act as PSD-95 scaffold to control albumin endocytosis, the glutamate transporter GLAST, and the metabotropic glutamate receptor mGluR5, in addition to activating Src phosphorylation and modulating the high density lipoprotein receptor SR-B1 that is crucial for cholesterol metabolism [44,45,46,47,48].

Further data mining at the Allen brain atlas, available online at: https://mouse.brain-map.org/ [49] retrieves evidence that *Atxn2l* mRNA has a particularly strong expression in cerebellar Purkinje neurons. Protein data in different cell types from the Human Protein Atlas, available online at: https://www.proteinatlas.org/ [50] demonstrate its prominent abundance in testis. Its moderate abundance in postmitotic neurons from the cortex, cerebellum, hippocampus, and caudate nucleus, as well as glia and muscle cells were detected by the Atlas antibody HPA041506 targeting ATXN2L at aa 456-547 (in the Uniprot Q8WWM7-1 sequence), which exhibits a single immunoblot band of predicted size and has siRNA-controlled specificity. However, it was not detected by Atlas antibody HPA043391, which targets human ATXN2L aa 572-644, but does not exhibit immunoblot signals of correct size and was not tested by siRNA, as can be seen within the Human Protein Atlas online at: https://www.proteinatlas.org/ENSG00000168488-ATXN2L/tissue/primary+data [50]. Subcellular analyses with both antibodies showed a diffuse cytosolic signal. Among cell lines, a particularly strong expression is detected under lymphoid differentiation, in good agreement with the importance of RNA surveillance for the innate and adaptive immune system. The upregulation of ATXN2L in cancer tissue is an unfavorable marker, in agreement with its published induction by EGF growth signaling [38]. A co-expression survey at the Coexpedia database, online available at: www.coexpedia.org [51] shows that mouse *Atxn2l* mRNA is regulated together with pathways of thin myocardium, open neural tube, improved glucose tolerance, immune defense, and cell cycle. The RNA binding spliceosome component *Srrm2* and the proline-rich inflammatory splicing factor *Prrc2a* show the highest co-expression score.

In a recently generated mouse with the aggregation of ATXN2 due to a polyglutamine (Q100) expansion knock-in [52], we observed a significant accumulation of ATXN2L peptides in cerebellar global proteome profiles from old animals. It was difficult to know if the impact of this anomaly has pathogenic or compensatory roles, and it was impossible to study it further in view of the unavailability of antibodies shown to be specific, of tagged recombinant constructs and mutant cells. We decided to start an exploratory project into the physiological functions of ATXN2L. Working at organism level, we generated novel *Atxn2l*-ablated mice to assess their phenotypes in growth, histology, and behavior, in comparison to our previous study of *Atxn2*-ablated mice [31]. Working at cell culture level, we examined the cellular phenotype of *Atxn2l*^−/−^ mouse embryonal fibroblasts (MEF), and whether the regulation of human *ATXN2L* and *ATXN2* mRNA levels is similar in response to stressors for neural cells. Overall, the data indicate that ATXN2L is much more important than ATXN2 for growth at early embryonal age, particularly in females. *Atxn2l* exon 5-8 deletion triggered a growth deficit phenotype rather than the weight excess observed in mice with *Atxn2* exon 1 deletion. Apparently, ATXN2L has a similar function as ATXN2, which is not only transcriptionally induced upon deprivation from nutrients and especially lipids, but also upon exposure to excess glucose or amino acids. Hence, we believe that elucidating the native role of ATXN2L throughout development and in response to stress provides important insights into the complementary spatio-temporal roles of ATXN2-ATXN2L and will further our understanding of neurodegenerative pathomechanisms.

## 2. Results

**Figure 1:**
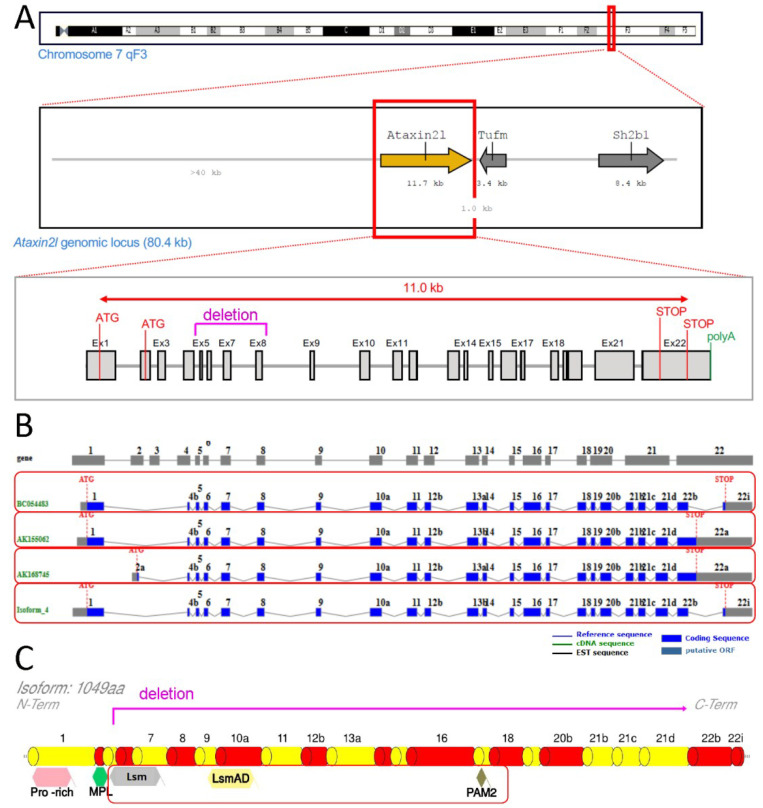
Scheme of genetic ablation within *Atxn2l*, with its splicing and translation effects. Ataxin-2-like (**A**) at the DNA level with neighbor genes *Tufm* and *Sh2b1*, indicating the deletion region in letters and lines of magenta color (also showing alternative translation start codons (ATG), structure from exon 1 to exon 22 with introns, alternative translation STOP codons, start of poly(A) tail); (**B**) at the mRNA level with alternatively spliced isoforms in separate red boxes; (**C**) at the protein level with relevant known sequence motifs (pro-rich refers to proline-rich-domain, MPL motif was reported by Meunier et al. 2002), highlighting the phylogenetically conserved Lsm, LsmAD and PAM2 motifs in a red box (deletion and frameshift are limiting translation to an N-terminal fragment until magenta line).

We obtained a null allele of murine *Atxn2l* via Crispr/Cas9-mediated deletion between two sgRNA sites flanking the exons 5-8 of the gene (outsourced work by TIGM, College Station, TX, USA) (see Figure 1).

**Figure 2:**
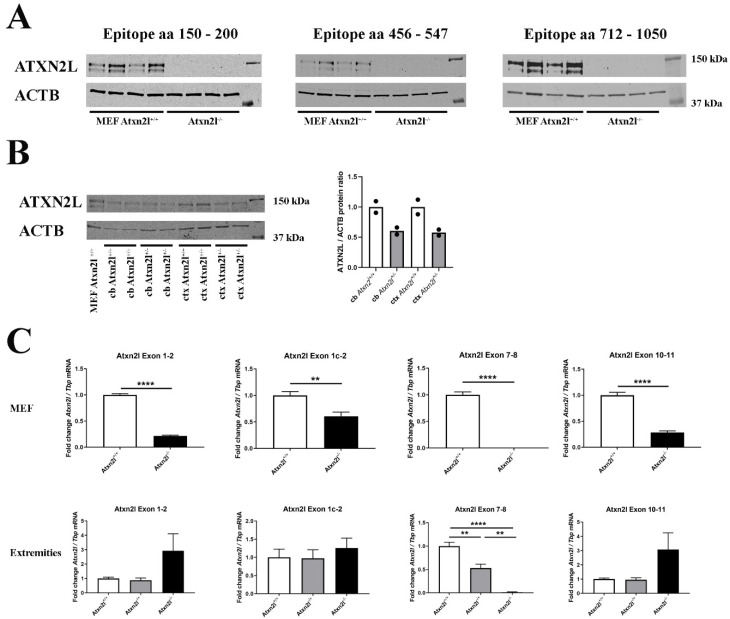
(**A**) Protein abundance of 3 different epitopes spanning ATXN2L in control *Atxn2l^+/+^* and homozygous *Atxn2l^−/−^* MEF (*n* = 4vs4). The protein was completely absent in three assessed epitopes from residue 150 until the most C-terminal region (aa: amino acid). (**B**) Protein abundance of ATXN2L in heterozygous *Atxn2l^+/−^* cerebellum (cb) compared to wildtype littermates, or in heterozygous *Atxn2l^+/−^* cortex (ctx), in comparison to one wildtype *Atxn2l^+/+^* MEF line. MEFs exhibit two specific bands of ATXN2L, in contrast to brain tissue where the larger band dominates. The overall ATXN2L levels were decreased to approx. 50% in heterozygous tissues (*n* = 2). Detection was done for the epitope aa 712-1050. (**C**) In *Atxn2l^−/−^* MEF (upper row, *n* = 4vs4), RT-qPCR showed that mRNA expression of *Atxn2l* was absent at the boundary between exons 7-8 that was genetically deleted, and significantly reduced at the boundaries of exons 1-2 and 10-11, possibly due to nonsense-mediated RNA decay. In tissue from fore-/hind-limbs [lower row, 3 wildtype (*Atxn2l^+/+^*, white bars), 4 heterozygous (*Atxn2l^+/−^*, grey bars), 2 *Atxn2l*-null embryos (*Atxn2^−/−^*, black bars)], the *Atxn2l* mRNA expression measured at the boundary of exons 7-8 was again absent in *Atxn2l^−/−^* and reduced by 50% in *Atxn2l^−/+^* mice, whereas 5′-upstream and 3′-downstream from the deletion the increased *Atxn2l* expression level of null mutants suggested transcript upregulation efforts either via the *Atxn2l* promoter or via altered mRNA stabilization/degradation to compensate the loss of protein function. The exon 1c-2 assay detects an alternatively spliced isoform. Asterisks represent significance (*p* < 0.01 **, *p* < 0.0001 ****).

The absence of ATXN2L protein epitopes from residue 150 until residue 1050 was confirmed in *Atxn2l*^−/−^ mouse embryonal fibroblast (MEF) (Figure 2A). ATXN2L abundance was higher in cerebral cortex than in cerebellum. While MEFs showed two specific ATXN2L bands in immunoblots, in brain tissue the larger band dominated (Figure 2B). The separate quantification of several *Atxn2l* mRNA exon junctions, namely 7-8 to represent the deleted region, 10-11 further downstream, and 1-2 upstream versus the alternatively spliced 1c-2 suggested the *Atxn2l*^−/−^ limb cells to sense the loss-of-function and either trigger the *Atxn2l* promoter or minimize the *Atxn2l* mRNA decay, thus maximizing the levels of the main *Atxn2l* mRNA variant. This feedback did not occur in MEF lines that are maintained in nutrient abundance (Figure 2C), suggesting that deprivation of overall energy or specific nutrients modulates *Atxn2l* transcription.

While the heterozygous mice bred normally, a lethality at the embryonic/prenatal stage was observed for the homozygous mutants. There was also reduced viability of heterozygous mice, particularly for females at the prenatal stage (see Table 1 and Table 2).

The dissection at different stages of pregnancy showed the death of homozygous mutants to have mostly occurred by embryonic day E14-16. Female *Atxn2l*^−/−^ embryos were more severely affected, most of them did not reach E11 (Table 2). These observations were in good agreement with reports in *C. elegans* worms that embryonal patterning is arrested, stem cell proliferation is reduced, and germline abnormally masculinized upon depletion of the ortholog ATX-2 [53,54]. Interestingly, at the 2-4 cell stage of bovine embryos, a 2.4-fold expression difference of *Atxn2l* was recently observed to correlate with fertility and that the knock-down of *Atxn2l* enhanced the development of blastocysts [55].

In the male homozygotes surviving beyond E11, there was a weight loss of 25% on average (Table 3). 

**Figure 3:**
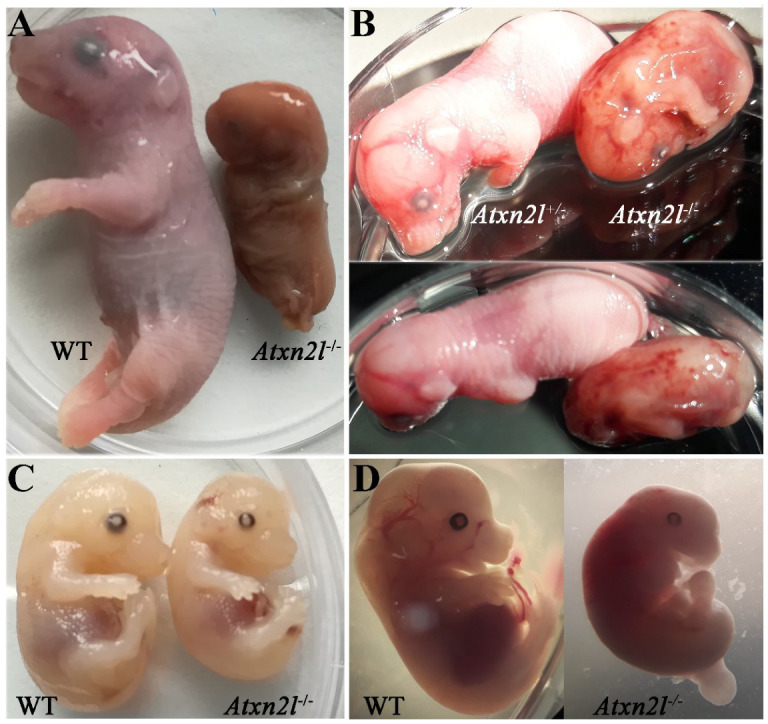
Comparison of *Atxn2l*^−/−^ embryos with their littermates, at different gestational stages. Embryonal days reflect approximate values. (**A**) The only *Atxn2l*^−/−^ mouse (male) identified at E20 in comparison to a male WT brother. (**B**) An *Atxn2l*^−/−^ mouse (male) reaching E16, exhibiting retarded development, and decreased weight, in comparison to the heterozygous *Atxn2l*^+/−^ female sibling. The petechial bleeds were not seen in other null mutants. (**C**) An *Atxn2l*^−/−^ mouse (male) at E15-16 with liver size and blood filling similar to male WT brother. (**D**) An *Atxn2l*^−/−^ female embryo at E13 with retarded growth and development and its male WT sibling.

Only one dead male *Atxn2l*^−/−^ embryo was identified at E20 (Figure 3A, right side), exhibiting disintegrating tissue surrounded by dense liquid as expected during resorption processes [56]. An *Atxn2l*^−/−^ embryo at E16 was observed to display retarded growth (Figure 3B, right side). The *Atxn2l*^−/−^ embryos were not pale, had livers of usual size, and showed normal blood-filling of liver tissue (Figure 3C, right side), so there was no evidence for the cardio-vascular or blood circulation deficits that are frequent causes of early or mid-gestational lethality [57]. Strong developmental differences between *Atxn2l*^−/−^ versus WT littermates (Figure 3D) sometimes amounted to several Theiler stages [58,59,60]. No selective organ affection in gross morphology was noticed during embryogenesis (Figure 3A–D), so an evaluation by high-resolution episcopic microscopy [61] was deemed unnecessary. In general, many fetal sacs in utero were found to contain only placenta rests and a fluid-filled cavity.

**Figure 4:**
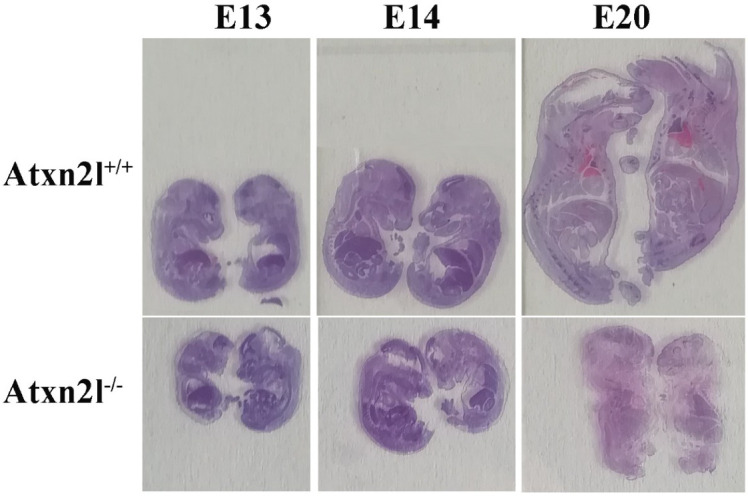
Evident growth/weight phenotype: *Atxn2l*^+/+^ embryos with *Atxn2l*^−/−^ littermates (WT above, homozygote mutants below, E13 on left side, E14 in center, E20 on right side).

Histological analyses at different embryonic stages from approximately E13 via E14 to E20 (Figure 4) confirmed that the growth deficit is generalized throughout the embryos.

The most frequent causes of early/mid-gestation embryonic lethality before E14 include deficient placentation, followed by blood vessel/heart, skeleton/joint and nervous system malformations [62]. In *Atxn2l*^−/−^ embryos, no obvious phenotypes apart from developmental delay were detected upon hematoxylin/eosin (H&E) staining of placenta (Supplementary Materials Figure S1), heart, and bones. Interestingly, mice with genetic ablation of the EGF receptor, which was observed in cell lines to influence *ATXN2L* expression, also exhibit a mid-gestational lethality. However, they display reduced placenta size [63,64] in contrast to the normal *Atxn2l*^−/−^ placentas. Mid-gestation embryonic lethality was recently also documented for factors of endocytic uptake, such as DENND1A and VPS54 [65,66]. 

**Figure 5:**
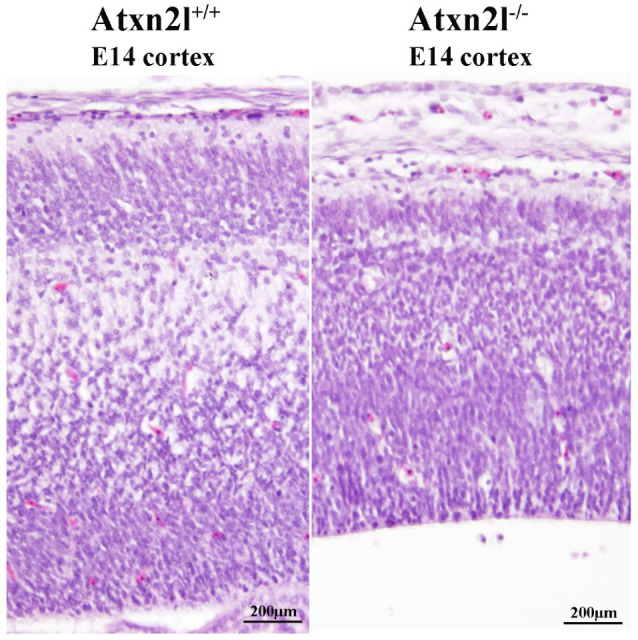
H&E histology at E14 showed good separation of neuron layers in the cortex of *Atxn2l*^+/+^ brain (left); in littermate *Atxn2l*^−/−^ mice (right), less lamina definition and many neurons were observed with nuclear condensation, which that is indicative of apoptosis; the diameter of brain cortex was also thinner.

In *Atxn2l*^−/−^ embryos, preferentially the nervous tissue showed frequent apoptosis, together with reduced cortical layer formation (Figure 5). Similar mid-gestation lethality with abnormal development of the brain cortex was observed, e.g., upon deficient methylation of eukaryotic mRNAs [67]. Neurons more than other cells have a need to modify the 3′-untranslated regions of mRNAs via alternative polyadenylation and microRNA repression [68,69] in a pathway that involves the ATXN2/ATXN2L indirect interactor TDP-43 and stress granules.

In view of the postnatal survival of *Atxn2l*^−/+^ mice, a cohort of 12 WT animals and 12 heterozygous littermates was aged until 12 months and assessed every 3 months regarding survival, growth, and locomotor behavior. This preliminary survey found no increased mortality or weight loss, and detected no prominent deficits in spontaneous movements in the open-field paradigm or in muscle coordination under stress on an accelerating rotarod (Supplementary Materials Figure S2).

**Figure 6:**
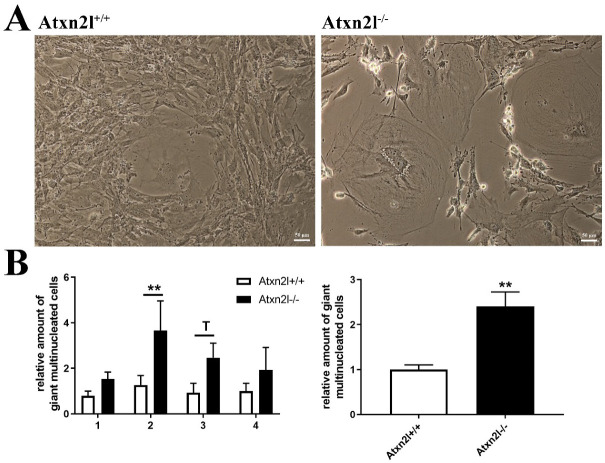
(**A**) Cell culture images of *Atxn2l*^+/+^ and *Atxn2l*^−/−^ MEF illustrating the increased number of giant multinucleated cells in the absence of *Atxn2l*. (**B**) Quantification of giant cells for 4 different age- matched littermate lines with 3 technical replicates each. The diagram on the left side shows the increase with consistency for the individual MEF lines 1-4, while the diagram on the right side represents the overall increase across all lines. Statistical testing was done by two-way ANOVA and t-test with Welch’s correction, respectively, significance levels were illustrated by ** for *p* < 0.01, by T for 0.05 < *p* <0.1.

Analyzing cell growth in four ATXN2L-null MEF lines versus four sex-matched WT littermates that were derived from dissected embryos, we noticed the presence many multinucleated giant cells. This cell type occurs in primary cell cultures when nuclear division is not followed by cytokinesis [70]. In ATXN2L-null MEF lines, their frequency was consistently increased, with a >2-fold effect size across all lines (Figure 6A,B). Cholesterol is necessary for cytokinesis, its deficiency induces polyploid cell formation, so it is noteworthy that cholesterol anomalies are well documented in patients and mice with ATXN2 mutations [31,71,72,73]. In addition, the furrow progression during cytokinesis depends on receptor tyrosine kinase activation of SRC and on vesicle endocytosis, where ATXN2 is known to play a modulating role [9,10,74,75,76].

**Figure 7:**
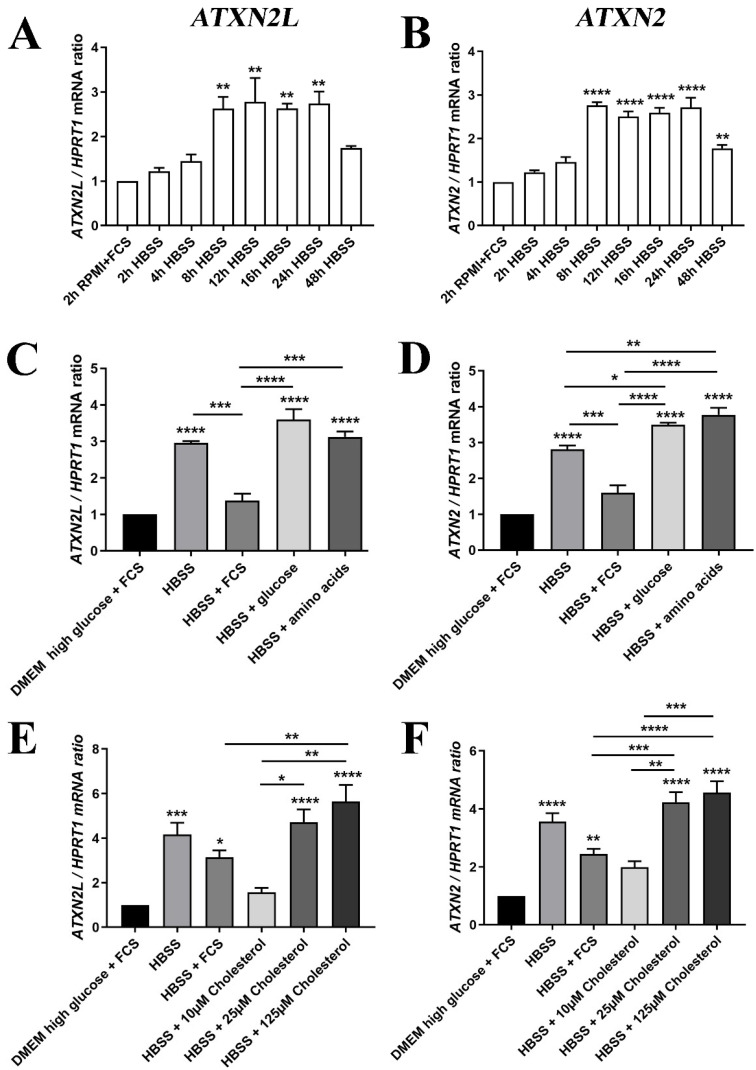
(**A**) The mRNA expression of *ATXN2L* in human SH-SY5Y neuroblastoma cells was induced by nutrient deprivation in HBSS medium over 48 h with low glucose/no amino acids, in the absence of lipids normally supplied via FCS, similar to the expression of (**B**) *ATXN2* mRNA. (**C**) For the induction peak at 24 h, the expression upregulation of *ATXN2L* was partially prevented upon supplementation of 10% FCS, while glucose or amino acids alone had no rescue effect; (**D**) FCS addition also diminished *ATXN2* expression, while glucose and amino acid administration enhanced the expression further; (**E**) The HBSS induction of *ATXN2L* was reduced at least as much by low cholesterol concentrations as by FCS, while higher dosage of cholesterol elicited even stronger upregulations. (**F**) This effect was similarly observed for *ATXN2.* A-D: *n* = 3; E/F: *n* = 8. Statistical testing was done by one-way ANOVA, significance levels were illustrated by asterisks: * *p* < 0.05; ** *p* < 0.01; *** *p* < 0.001; **** *p* < 0.0001. Comparison was always in respect to nutrient abundant control condition, if not stated otherwise.

Then, we assessed whether the deprivation of nutrients, such as glucose and lipids, indeed alter the expression of *Atxn2l*, as was previously observed for its homolog *Atxn2* [31]. The human neuronal cell line SH-SY5Y, first cultured in high-glucose DMEM medium with fetal calf serum (FCS) supplement, was switched to the starvation medium HBSS without FCS for a time course of 48 h. The sudden exposure to conditions of low glucose, no amino acids, no FCS (containing growth factors/apolipoproteins with lipids/transferrin with iron, etcetera) triggered a phasic induction that peaked at 8–24 h with a 3-fold *Atxn2l* mRNA increase, similar to the induction of *Atxn2* (Figure 7A,B). Preliminary testing in MEF cells suggest that RNA levels of *Atxn2* levels decreased slightly upon to stress by a toxic RNA-analogon, Poly(I:C), while *Atxn2l* failed to respond (Supplementary Materials Figure S3E–H). These MEF analyses provided no evidence that the depletion of ATXN2 triggers compensatory dysregulation of its homolog Ataxin-2-like at mRNA or protein level, or that conversely the depletion of ATXN2L triggers expression or abundance changes of Ataxin-2 (Supplementary Materials Figure S3E–J). 

Further analysis of individual nutrients demonstrated that the transcriptional induction of *ATXN2L* in HBSS starvation medium could be diminished by the supplementation with FCS (which contains components such as insulin, transferrin, and apolipoproteins), and by low level cholesterol administration, two effects that were similarly observed for *ATXN2*. This was in contrast to supplementation with excess glucose, amino acids, or excess cholesterol that were unable to downregulate *ATXN2L* or further enhanced its levels (Figure 7C,E). The increased transcription of *ATXN2* upon glucose and amino acid addition was significant, as well as the induction by cholesterol excess (Figure 7D,F).

Further analyses about components of FCS that could be responsible for the rescue effect in the induction of *ATXN2L* are shown in Supplementary Materials Figure S3. Different cell culture media were used as basal media (DMEM having the highest abundance of nutrients, MEM containing fewer nutrients such as vitamins or amino acids, and HBSS containing no nutrients apart from low glucose amounts). The supplements included SPITE (a mixture of sodium selenite, sodium pyruvate, bovine insulin, human transferrin, and ethanolamine), ITS (a mixture of bovine insulin, human transferrin, and sodium selenite), and SITE+3 (containing analogous components as SPITE, but 5 mg/mL BSA, linoleic acid and oleic acid as a lipid energy source instead of pyruvate). However, none of these supplements was sufficient for a significant rescue of the starvation-triggered *ATXN2L* induction, to a degree that approached FCS. The comparison of SPITE versus SITE+3 supplementation suggested that fatty acid administration is more helpful than pyruvate for the reduction of *ATXN2L* and *ATXN2* mRNA levels (Supplementary Materials Figure S3C,D). Overall, these in vitro observations confirm the notion derived from limb tissues in vivo and from MEF cells, that either the promoter activity of the Ataxin-2-like gene is upregulated or the *Atxn2l*/*Atxn2* mRNA decay is downregulated during periods of growth deficits and then this regulation can be reversed upon availability of specific nutrients. In this context, it is noteworthy that the RegRNA2.0 webtool predicts both transcripts to contain AU-rich elements in their 3′-untranslated region, which are known to modulate the stability of selected mRNA according to growth needs. It is also relevant to mention that the promoters/enhancers of human Ataxin-2/Ataxin-2-like share 476 transcription factor binding sites, according to predictions found at the GeneCards website, including stress-response regulator families that are strongly implicated in neurodegeneration, such as ATF1/2/3/4/7, E2F1/5/6/8/ELF1/3/4/ETS1/ETV1/4/5/6, HNRNP-H1/K/L/LL/UL1, or IRF1/2/3/4.

A dysregulation of ATXN2L levels upon depletion of its ortholog ATXN2 was not observed in MEF cells, nor a converse dysregulation of ATXN2 upon depletion of ATXN2L, neither at protein nor at mRNA level (Supplementary Materials Figure S3E–J). As mentioned in the Introduction however, unpublished global proteome mass spectrometry data in our lab documented a significant increase of ATXN2L in the brain of *Atxn2*-CAG100-knockin mice. This finding may therefore be specific to the CAG-repeat expansion mutation and be caused by the polyQ aggregation process in stress granules, where ATXN2 and ATXN2L have common protein interactors such as DDX6 and may co-precipitate.

## 3. Discussion

ATXN2L was described as direct RNA-binding protein with sequence homology to nuclear spliceosome factors [37,77]. Its alternatively spliced isoforms may interact with tyrosine kinase receptor signaling at the plasma membrane [36]. The constitutive isoform of ATXN2L was mainly characterized for its role as surveillance factor during transient periods of cell damage, which promotes the formation of stress granules in the cytosol [34]. Now, our ATXN2L-deficient mouse data demonstrate that homozygous loss of its RNA-interaction domains is incompatible with embryonic development from early stages. Their absence delays the cytokinesis stage of cell division and impairs the layer formation and survival of brain neurons. 

Human data agree with selective roles of ATXN2L for the growth process, the nervous system, and immunity. Genome-wide association studies (GWAS) of single nucleotide polymorphism (SNP) alleles versus phenotypes in healthy populations (summarized at the NHGRI-EBI Catalog of human genome-wide association studies, online available at: https://www.ebi.ac.uk/gwas/ [78] demonstrated significant effects in several independent studies of the *ATXN2L* gene locus. Its impact on growth was reflected by associations with body mass index (in diverse reports for different polymorphisms *p* = 4e^−29^; 2e^−15^; 9e^−15^; 2e^−13^; 3e^−8^; 2e^−7^) [79,80,81], body weight (*p* = 2e^−8^) [81], and waist circumference (*p* = 4e^−8^) [81]. Its impact on the nervous system was reflected by associations with cognitive function (*p* = 8e^−35^) [82], grip strength (*p* = 1e^−25^) [83], intelligence (*p* = 1e^−19^; 2e^−9^; 1e^−8^; 2e^−8^; 4e^−8^) [84,85,86,87], and educational attainment (*p* = 4e^−14^; 7e^−11^; 1e^−7^; 1e^−6^) [85,88,89]. Its impact on immunity was reflected by associations with albumin/globulin ratio (*p* = 6e^−10^) [90], inflammatory bowel disease (*p* = 2e^−9^) [91], and pediatric autoimmune diseases (*p* = 6e^−7^) [92].

Our studies of the expression regulation of *Atxn2l* and *Atxn2* by diverse nutrients indicate a strong similarity in their role, with a transcriptional induction being triggered by nutrient deprivation as well as by an excess of cholesterol and glucose (both of which have a role for plasma membrane flexibility/stickiness), while the transcription was reduced upon the supplementation of FCS. Given that the depletion of the ATXN2L ortholog in flies is neuroprotective, there may be therapeutic value in the knowledge that its expression can be downregulated in mammalian cells by the abundance of specific nutrients. The observations here may be relevant for the treatment of ALS, where hypercaloric feeding was shown to be beneficial, and evaluations on the relative contribution from carbohydrates versus lipids are ongoing [93,94]. An experimental artifact was observed upon nutrient deprivation and upon Poly(I:C) stress in vitro: Although the induction strength of *Atxn2* and *Atxn2l* showed limited variance during repeated experiments by a student with a SH-SY5Y aliquot and a specific FCS batch, it could however show quite different fold-changes months later in different hands using a separate SY-SY5Y stock with another FCS batch. It remained unclear to what degree passage number, clonal mutation drift or handling variance played a role here (compare Figure 7C vs. E, and Figure S2 F vs. H). 

Publicly available expression data on *Atxn2l* and *Atxn2* by RNA sequencing and further processing at the Broad Institute within the Genotype-Tissue-Expression project database, online available at: https://gtexportal.org/home/ [95] with normalization to transcripts per million reads (TPM) confirm that *Atxn2* and *Atxn2l* are both preferentially expressed in cerebellar hemispheres and total cerebellum (median TPM value = 30/33 vs. 184/229, respectively), in comparison with other regions of the nervous system. Interestingly, our observations on sex differences in the phenotype of null mutants are also reflected by these public expression data: they document that *Atxn2* expression is stronger in most female organs (36/31/30/29/28/24 TPM in ovary/uterus/endocervix/FallopianTube/ectocervix/vagina, respectively) than in cerebellum. In contrast, *Atxn2l* shows the highest expression in testis tissue (TPM 296), as opposed to the average expression in female organs (e.g., ovary TPM 124). Overall, *Atxn2l* in cerebellum shows a 6-fold higher expression than *Atxn2*, and the preferential expression of *Atxn2l* in male testis contrasts with the pronounced expression of *Atxn2* in several female sexual organs. Given that the biosynthesis of all sex hormones starts from cholesterol, the effects of ATXN2L and ATXN2 on the cholesterol homeostasis may account for the sex difference of *Atxn2l*-null phenotypes, the increased formation of giant multinucleated MEF cells, and the expression regulation by cholesterol. It is important to note that *Atxn2*-mutations were already observed to affect cholesterol homeostasis and trigger gender-dependent or hormonal effects [31,73,96,97]. Giant multinucleated cells can be due to increased mTOR activity [98,99,100]. Given that ATXN2 was reported to inhibit mTOR in yeast, nematodes and mice; also the absence of ATXN2L might lead to enhanced mTOR signaling and, in this way, increase the appearance of multinucleated giant cells in MEF cultures. These phenotypes may be downstream effects of the reported protein interaction between ATXN2L and NHERF2 as a known modulator of cholesterol metabolism and Src-dependent phosphorylation signals for growth. While a gene duplication event in mammals led to the coexistence of two homologous genes, in plants a coexistence of four homologous genes from the ataxin-2 family raises the possibility that mTOR-dependent growth can be regulated by each of them in response to different stressors or to different nutrients, via an RNA-binding mechanism.

Clearly, the regulation of *Atxn2l* and *Atxn2* mRNA levels by nutrients is almost parallel in our cell culture analyses, so it seems paradoxical that our null mutant of ATXN2L has a growth deficit phenotype, while the null mutant of ATXN2 was found by two independent teams to show a converse nutrient excess phenotype [31,32]. An important difference in the recombinant design of these mutants should be noted here. The KO of *Atxn2* was targeting exon 1 and removed all of the protein domains, while the ablation in *Atxn2l* targeted exons 5-8, with a resulting shift of reading frame downstream. In the mutant ATXN2L, the N-terminal Pro-rich domain across residues 4-61 and the MPL interaction domain across residues 96-119 would still be synthesized as a fragment, the available commercial antibodies cannot detect this N-terminal region. In view of the increase of *Atxn2l* non-targeted exons in the limb tissue of homozygous mutants (Figure 2B), this hypothetical fragment could exist in overdose. The excess amounts of such an N-terminal fragment would interact with plasma membrane receptors and with the endocytosis machinery. In analogy to the published observations for ATXN2 overexpression, it is expected to impair trophic uptake mechanisms [9]. At the same time, the absent middle and C-terminal part of ATXN2L would fail to interact with RNAs in the appropriate manner. Overall, our mouse mutant might represent not only a loss of the C-terminal functions of ATXN2L; it is conceivable that it also involves a toxic gain of N-terminal functions of ATXN2L. Perhaps this speculation provides a clue why our mouse mutant has this unexpected phenotype, which is converse to *Atxn2*-KO mice. 

In the future, antibodies against the N-terminal portion of ATXN2L should be generated to obtain evidence regarding this doubt, and a new ATXN2L mutant that targets exons 1-4 would also help to clarify this issue. It will be crucial to generate conditional mutants where the postnatal role of ATXN2L for tissues such as the aging brain can be studied selectively. Moreover, it is interesting to note that commercial antibodies such as HPA041506/PA5-59601 in U2OS cells showed only the band of predicted size, and our immunoblots in brain tissue also detected this putative full-length band, while we observed two ATXN2L bands with three different antibodies in MEF cells, and also the A301-369A and 24822-1-AP antibodies detected a smaller band in HeLa/HEK293T and Jurkat cells, according to manufacturer datasheets. Thus, further studies will be necessary to understand the usage of the two alternative start codons and of the alternative splice variants in various tissues/cell populations and after different stressors. Clearly, our data indicated that the depletion of ATXN2 or ATXN2L in MEF cells fail to trigger a compensatory upregulation of the other homolog, so the unpublished accumulation of ATXN2L in *Atxn2*-CAG100-KIN brain appears to be an expansion-driven effect and ATXN2L immunoreactivity should be assessed within the Q100-ATXN2 aggregates. Since TDP-43 is essential for embryonic development in mouse and its aggregation drives the decisive toxicity for motor neuron degeneration, it is conceivable that ATXN2L—a factor that is also essential for embryonic development—could co-aggregate with ATXN2 and drive toxicity in cerebellar Purkinje neurons where it has selective abundance.

## 4. Materials and Methods

### 4.1. Generation, Breeding, Maintenance, and Dissection of Atxn2l^−/−^ Mice

To specifically target sites flanking exons 5-8 of the *Atxn2l* gene, this pair of sgRNAs was used: upstreamGRNA085CCT (atgtggcagggtcagtatcag), downstreamGRNA02CCC (gttacctggataccaagct). To distinguish the long wildtype from the short mutant allele, primers *Atxn2l*-F (gagagtgtgtgttgggtgac), *Atxn2l*-F2 (gaagcacagtgctgttcagc), and *Atxn2l*-R (gtaatttgcagcaacagtagagc) were employed. Heterozygous breeders were shipped, crossed at the ZFE animal facility of Goethe University Medical Faculty in Frankfurt as described [101] and health-monitored with sentinel animals at quarterly intervals. Congenic heterozygous mice were then crossed among them. The studies were ethically approved by Regierungspräsidium Darmstadt, code V54-19c20/15-FK/1083, on 11 March 2019. 

### 4.2. Genotyping

DNA was extracted from adult ear punches or embryo tail biopsies for genotyping by incubating at 95 °C in 100 µL of 25 mM NaOH/0.2 mM EDTA for 30 min before neutralizing with 100 µL 40 mM Tris–HCl, pH 5.0. For the subsequent genotyping reactions, 1 µL of lysed tissue sample was used per reaction. The *Atxn2l* alleles were amplified by PCR with the primers Atxn2l-F (GAGAGTGTGTGTTGGGTGAC), Atxn2l-F2 (GAAGCACAGTGCTGTTCAGC) and Atxn2l-R (GCTCTACTGTTGCTGCAAATTAC), the PCR products 217 bp and 309 bp corresponding to *Atxn2l* wt and *Atxn2l* mutant respectively were separated in 1.5% agarose gel.

For the determination of the sex, primers Rmb31F (CACCTTAAGAAGCCAATACA) and Rbm31R (GGCTTGTCCTGAAAACATTTGG) were used to yield a 269 bp product from X chromosome and a 353 bp product from the Y chromosome [102].

### 4.3. Behavioral Tests 

Behavioral tests were done as described in [103]. Twelve *Atxn2l*^+/+^ and twelve *Atxn2l*^+/−^ male mice were aged and assessed at ages between 3 months and 12 months. 

### 4.4. Generation and Cell Culture of MEF Cells 

MEF were derived from *Atxn2l*^−/−^ embryos at day 14-16 and their littermate *Atxn2l*^+/+^ controls. As previously described [104], the skin of the embryos was dissected, homogenized, and trypsinized for 10 min at 37 °C. The cells (passage 2-3) were cultured in DMEM, containing 4.5 g/L D-glucose, 1% L-glutamine, 1% Penicillin/Streptomycin and 10% FCS. *Atxn2*^−/−^ MEF cells were described before [9].

### 4.5. Poly(I:C) Treatment 

*Atxn2*^+/+^ and *Atxn2*^−/−^ MEF (3 vs. 3 cell lines), as well as *Atxn2l*^+/+^ and *Atxn2l*^−/−^ MEF (4 vs. 4 cell lines) were treated with 1 µg/mL Poly(I:C) (Invivogen) for 16 h with subsequent RNA extraction and RT-qPCR [105]. 

### 4.6. RNA Extraction and Expression Analysis

RNA extraction from limbs and MEF was performed with TRIzol Reagent (Sigma Aldrich) according to the user manual. RNA from cell culture experiments with SH-SY5Y cells was performed with Extractme Total RNA Kit (7Bioscience EM09.1-250). Synthesis of cDNA from 1 µg of total RNA template was performed with the SuperScript IV VILO kit (ThermoFisher) according to the manufacturer’s instructions. cDNA from 25 ng total RNA was used for each RT-PCR reaction with 0.5 µL TaqMan^®^ Assay, 5 µL FastStart Universal Probe Master 2x (Rox) Mix and ddH_2_O up to 10 µL of total volume. The mouse-specific TaqMan^®^ Assays utilized for this study: *Atxn2l* (Mm00805548_m1, Mm00805539_m1, Mm01165459_m1, Mm01276208_m1), *Atxn2* (Mm01199894_m1), and *Tbp* (Mm00446973_m1).

### 4.7. Immunoblotting

Protein was isolated with RIPA buffer as described before [106]. Samples with 20 µg protein were separated on 8% SDS gels and blotted on nitrocellulose membranes. Membranes were incubated overnight at 4 °C with the following antibodies: ATXN2L (Bethyl A301-369), ATXN2L (Invitrogen PA5-59601), ATXN2L (Proteintech 24822-1-AP), ATXN2 (Proteintech 21776-1-AP) or beta-Actin (Sigma A5441), and for 1 h with the respective secondary antibodies (Li-Cor). Fluorescence was detected with the Li-Cor Odyssey Classic Instrument and bands were analyzed with Image Studio Lite, Version 5.2.

### 4.8. Sections and Staining

All tissue specimens were fixed in 4% paraformaldehyde (PFA) for at least 48 h. All specimens were embedded in paraffin and aligned as displayed on the respective images (see Figure 4 and Figure 5 and Figure S1). The 4-µm thick sections were made using a microtome. Slides were stained with hematoxylin and eosin according to standard protocols. Images were taken using an Olympus BX50 microscope and an Olympus DP72 camera.

### 4.9. Microscopy and Cell Counting of MEF 

175.000 MEF cells were seeded in 6-well plates and grown overnight (*n* = 4 *Atxn2l*^+/+^ vs. 4 *Atxn2l*^−/−^ lines in 3 technical replicates each). For each well, 5 random areas were microscopically visualized (Leica) and all giant cells were counted. Means for each cell line was calculated and statistically evaluated. Images were taken with a Nikon microscope after changing the cell culture medium to PBS. 

### 4.10. Cell Culture Experiments 

SH-SY5Y cells were purchased from ECACC, and cultured as described [6] in Dulbecco’s Modified Eagle Medium (DMEM) containing 4.5 g/L D-glucose, 2 mM L-glutamine with 10% FCS. The cells were starved of trophic factors by incubating them in Hanks’ balanced salt solution (HBSS) for a range of different periods (time course) to determine when the cells show the strongest deprivation effect. In different experiments, HBSS was supplied with 10% FCS, glucose 4.5%, MEM non-essential amino acids solution 1×, or cholesterol (SIGMA L4646) at 10 µM, 25 µM, cholesterol 125 µM (Figure 7). For the experiments shown in Supplementary Figure S3, cells were incubated with the indicated basal media (DMEM, MEF, or HBSS), supplemented with SPITE medium supplement 1x (Sigma S5666), ITS liquid media supplement 1x (Sigma I3146), or SITE+3 liquid media supplement 1x (Sigma S5295). After 24 h, cells were collected and RNA was extracted with EXTRACTME (Blirt, Gdańsk, Poland). Synthesis of cDNA and RT-qPCR was performed as previously described with human-specific TaqMan^®^ Assay *ATXN2L* (Hs00944485_g1), *ATXN2* (Hs00268077_m1) and *HPRT1* (Hs99999909_m1). The numbers of cell lines used is stated in respective figure legends.

### 4.11. Statistical Analysis

All statistical tests were performed as unpaired Student’s t-test with Welch’s correction or one-way or two-way ANOVA using GraphPad Prism software version 7 after establishing that each population was normally distributed (one-sided Kolmogorov–Smirnov test). Figures display mean values and standard error of the mean (s.e.m.). Values of *p* < 0.05 were considered significant and marked with asterisks *p* < 0.05 *, *p* < 0.01 **, *p* < 0.001 ***, *p* < 0.0001 ****.

## 5. Conclusions

Overall, this first analysis of ATXN2L-depletion in mouse demonstrates its essential role for embryonic development, due to growth delays with prominent vulnerability of brain lamination, especially in females. A preferential affection of female embryos and an increased occurrence of multinucleated giant cells in MEF culture were observed, which may reflect a role of ATXN2L in cholesterol and hormone homeostasis. This mouse mutant will provide an important tool to dissect the role of ATXN2L domains, such as the impact of LSM and PAM2 motifs on RNA surveillance, which were ablated here, versus the effects of PRD and MPL interaction motifs on trophic endocytosis, during stress periods and aging. Since the data show ATXN2L to be more important than ATXN2 for cell growth, it will be highly interesting to study its role in neurodegenerative disorders due to RNA surveillance problems, such as ALS, where the depletion of mammalian ATXN2 and of the yeast/fly ortholog also of ATXN2L was already shown to be neuroprotective.

## Figures and Tables

**Table 1:** Postnatal genotype distribution for offspring from *Atxn2l*^+/−^ intercrosses.

**Observed**/Expected Number of Live Born Mice with Indicated Genotype				
	+/+	+/−	−/−	Number of offspring
Live born female	**50**/55	**77**/110	**0**/55	**127**/220
Live born male	**60**/55	**83**/110	**0**/55	**143**/220
Live born total	**110**/110	**160**/220	**0**/110	**270**/440

**Table 2:** Embryonic day E11-20 genotype distribution for offspring from *Atxn2l*^+/−^ intercrosses. Most lethality occurred before E11, with females dying before males.

**Observed**/Expected Number of Mice with Indicated Genotype				
	+/+	+/−	−/−	Number of offspring
Dissected embryos female	**48**/39	**57**/78	**5**/39	**110**/156
Dissected embryos male	**30**/39	**71**/78	**16**/39	**117**/156
Dissected embryos total	**78**/78	**128**/156	**21**/78	**227**/312

**Table 3:** Weight of embryos in mg directly after dissection at three different embryonal ages. *n*: *Atxn2l*^+/+^ = 1-2, *Atxn2l*^+/−^ = 4-6, *Atxn2l*^−/−^ = 1, E = embryonal day.

Weight of Embryos [mg]			
	+/+	+/−	−/−
Day E14–15	322	306	226
Day E15–16	447	444	313
Day E20	1472	1481	372

## Supplementary Materials

Supplementary materials can be found at https://www.mdpi.com/1422-0067/21/14/5124/s1.

## Abbreviations

°C	Degrees Celsius (temperature)
aa	amino acid
ACTB	beta-actin
Adnp	Activity-Dependent Neuroprotector Homeobox Protein
ALS	Amyotrophic Lateral Sclerosis
ANOVA	Analysis of variance
ARHGEF11	Rho Guanine Nucleotide Exchange Factor (GEF) 11
ATF1	Activating Transcription Factor 1
ATG	Start codon Adenine-Thymidine/Uracil-Guanine
ATX-2	the single nematode ortholog of mammalian Ataxin-2 and Ataxin-2-like
ATXN2	Ataxin-2
ATXN2L	Ataxin-2-like, aka: Ataxin-2-Related Protein, A2LP, A2RP or A2D
BDNF	Brain-Derived Neurotrophic Factor
bp	Base pair
BSA	Bovine serum albumin
Carm1	Coactivator Associated Arginine Methyltransferase 1
Cart1	Cartilage paired class homeoprotein 1 (aka Alx1)
Cb	Cerebellum
Cbp	cAMP-response element binding protein (Creb) binding protein (aka Crebbp)
cDNA	complementary Deoxyribo-Nucleic Acid
CID3	CTC-interacting domain 3 protein in *Arabidopsis thaliana*
CID4	CTC-interacting domain 4 protein in *Arabidopsis thaliana*
Cited2	Cbp/P300 Interacting Transactivator With Glu/Asp Rich Carboxy-Terminal Domain 2
Crispr/Cas9	Clustered Regularly Interspaced Short Palindromic Repeats/Crispr-associated protein 9
C-term	C-terminal end of the protein
Ctx	Cortex
DAPK1	Death-Associated Protein Kinase 1
DDX6	DEAD/H (Asp-Glu-Ala-Asp/His) Box Polypeptide 6 (RNA Helicase, 54kD)
DENND1A	DENN/MADD Domain Containing 1A (RAB35-activating guanine nucleotide exchange factor)
DMEM	Dulbecco’s modified essential medium
DNA	Desoxyribo-Nucleic-Acid chain
E	Embryonic development day
E2F1	E2F Transcription Factor 1
e.g.,	Exemplo gratia (for example, in Latin language)
EGF	Epidermal growth factor
ELF1	E74 Like ETS Transcription Factor 1
Ep300	E1A Binding Protein, Histone Acetyltransferase P300
EST	Expressed sequence tag
Ex1	Exon 1
FCS	Fetal calf serum
FTLD	Fronto-temporal lobar dementia
Foxj2	Forkhead Box Protein J2, a transcriptional activator
g	Gram
G3BP2	Ras-GTPase Activating Protein SH3 Domain-Binding Protein 2
GLAST GWAS	Sodium-Dependent Glutamate/Aspartate Transporter 1 (in astrocytes) Genome wide association studies
h	hour
H&E	Hematoxylin and Eosin staining
HBSS	Hank’s buffered salt solution
Het	Heterozygote
HNRNP	Heterogeneous Nuclear Ribonucleoprotein
Kb	kilo-base
IRF1	Interferon Regulatory Factor 1
ITS	Insulin-Transferrin-Selenium
KD	Knock-Down
KO	Knock-Out
L	liter
lncRNA	long non-coding RNA
Lsm	Like “Smith antigen” protein domain
LSM12	Homolog of yeast cytosolic LSM12 protein
LsmAD	Lsm-associated domain
mg	milliGram
mm	milliMeter
mM	milliMolar
µg	microGram
µL	microLiter
µM	microMolar
MEF	Mouse embryonal fibroblasts
MEM	Minimal Essential Medium
MGI mGluR5	Mouse Genome Informatics metabotropic Glutamate Receptor 5
MPL	Interactor domain with Myelo-Proliferative Leukemia gene, encodes Thrombopoietin receptor
mRNA	Messenger ribonucleic acid
mTORC1	Mechanistic “target of rapamycin” complex 1
Neat1	Nuclear Paraspeckle Assembly Transcript 1
NFATC2IP	Nuclear Factor of Activated T-Cells, Cytoplasmic, Calcineurin-Dependent 2 Interacting Protein
ng	nanoGram
NHGRI-EBI N-term	American National Human Genetics Research Institute & European Bioinformatics Institute N-terminal end of the protein
NUFIP2	Nuclear Fragile X Mental Retardation Protein 1 Interacting Protein 2
ORF	open reading frame
p	probability value for the occurrence of a given observation by chance
PABPC1	Poly(A)-binding-protein cytosolic 1
PAM2	Poly(A)-binding mediator domain 2
PBS	Phosphate buffered saline
PFA	Paraformaldehyde
poly(A) tail	adenosine-repeat at the 3′ end of mRNA
Poly(I:C)	Poly-Inosinic: Poly-Cytidylic acid chain
Pro	Proline
PRD	Proline-rich domain
PSD-95 PSP	Postsynaptic Density Protein 95 (aka DLK4, Disks Large Homolog 4) progressive supranuclear palsy (aka Parkinson plus)
Q, 22Q	glutamine, repeat of 22 consecutive glutamines
RA	Retinoic acid
RT-qPCR	Reverse-Transcriptase quantitative Polymerase Chain Reaction
S6R	Small ribosomal subunit protein 6
SCA1	Spinocerebellar Ataxia type 1
SCA2	Spinocerebellar Ataxia type 2
SDS	Sodium-Dodecyl-Sulfate detergent
s.e.m.	Standard error of the mean
Setd5	Histone-Lysine N-Methyltransferase, SET domain containing 5
SG	Stress granule
sgRNA	Single guide Ribo-Nucleic-Acid chain
Sh2b1	Pro-Rich, PH And SH2 Domain-Containing Signaling Mediator gene
SH3	Src-homology 3 domain
SH-SY5Y	Cell line subcloned from SK-N-SH line from 4-year-old female neuroblastoma patient
SITE+3	Selenite, Insulin, Transferrin, Ethanolamine +3
SLC9A3R2 SNP	Solute Carrier Family 9 (Sodium/Hydrogen Exchange) Member 3 Regulator 2 (aka NHERF2) Single nucleotide polymorphism
SPITE	Selenite, Pyruvate, Insulin, Transferrin, Ethanolamine +3
SR-B1 Src	Scavenger Receptor Class B Member 1 (aka CD36, Thrombospondin Receptor-Like 1) V-Src Avian Sarcoma (Schmidt-Ruppin A-2) Viral Oncogene
Tbp	TATA-binding factor of transcription
TDP-43	TAR (transcription active response element) DNA-Binding Protein 43 (encoded by *TARDBP*)
Tfap2a	Transcription Factor Activator-Protein-2 alpha
TIGM, TX	Texas Institute of Genomic Medicine, Texas, United States of America
TPM	Transcripts per million (expression unit as normalization method in RNA-sequencing)
Tufm	Tu Translation Elongation Factor, Mitochondrial gene
ULK1	Unc-51 Like Autophagy Activating Kinase 1
VPS54	Vacuolar Protein Sorting-Associated Protein 54 (endosomal factor for retrograde transport)
WT	Wild-Type
