# Supplementary material for: Mid-Gestation lethality of Atxn2l-Ablated Mice
Source: Int J Mol Sci. 2020 Jul 20;21(14):5124. doi: 10.3390/ijms21145124 (PMC7404131; doi:10.3390/ijms21145124)
Supplement: Supplementary file 1 [file ijms-21-05124-s001.pdf]

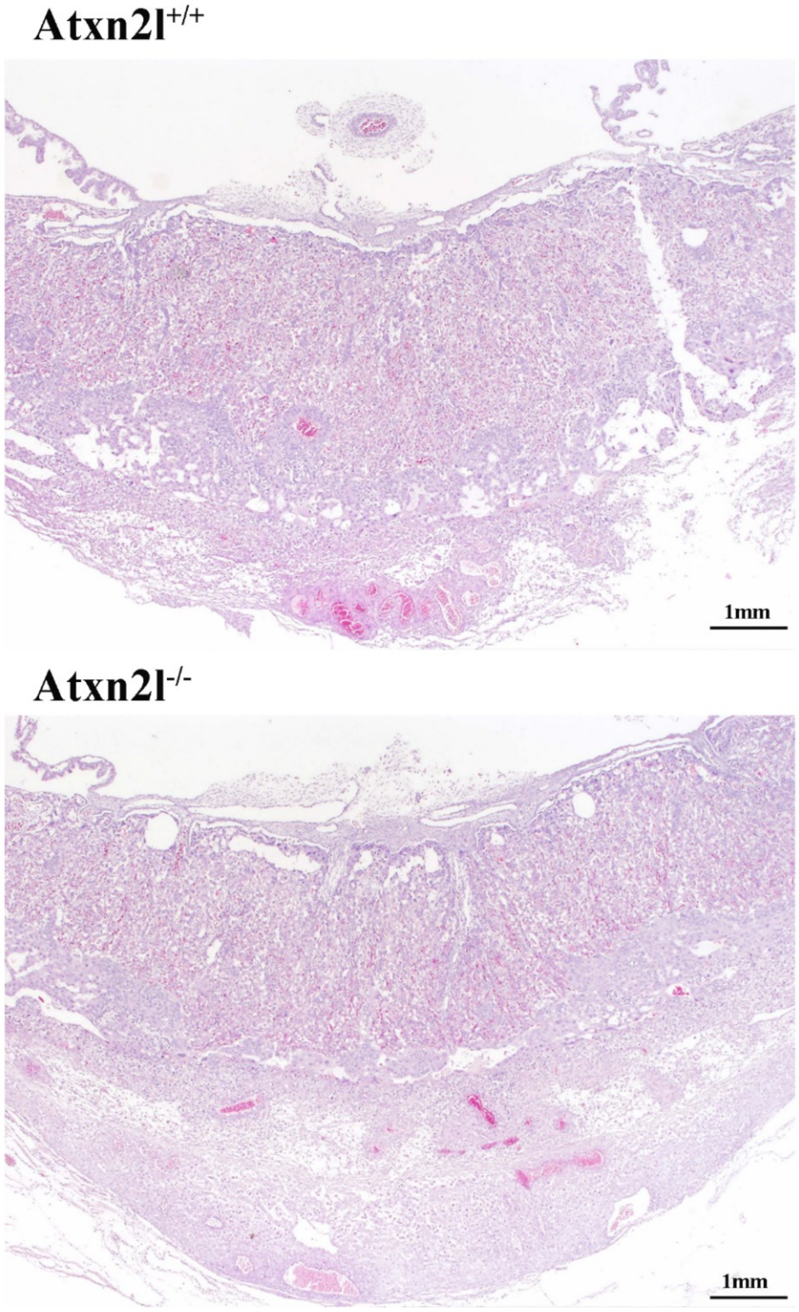

Figure S1. Histology (H&E stain) of placentas from an *Atxn2l*$^{+/+}$ embryo (upper image) and *Atxn2l*$^{-/-}$ (lower image) revealed no phenotype differences.

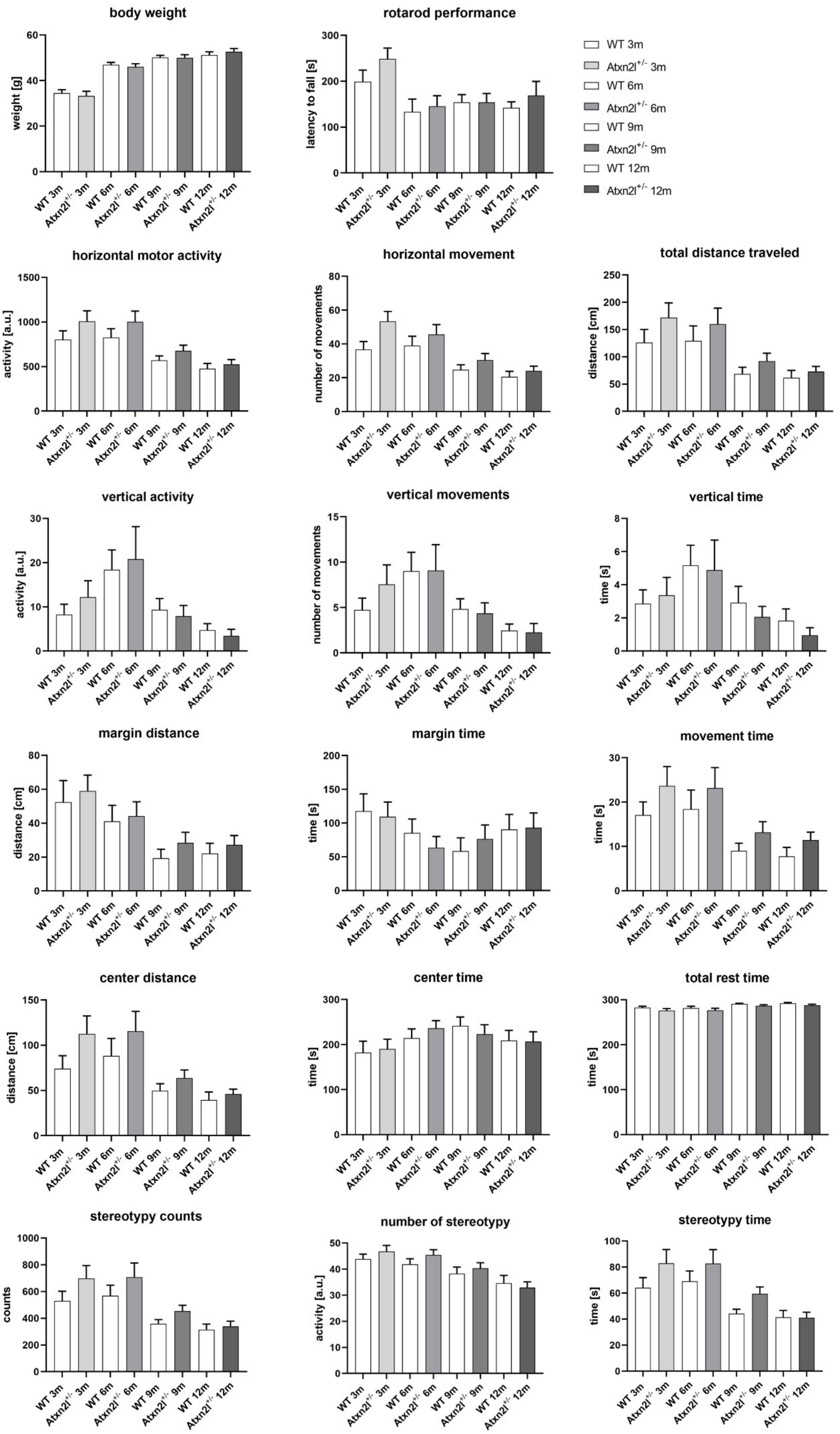
body weight
weight [g]
rotarod performance
latency to fall [s]
WT 3m
Atxn2l+/- 3m
WT 6m
Atxn2l+/- 6m
WT 9m
Atxn2l+/- 9m
WT 12m
Atxn2l+/- 12m
horizontal motor activity
activity [a.u.]
horizontal movement
number of movements
total distance traveled
distance [cm]
vertical activity
activity [a.u.]
vertical movements
number of movements
vertical time
time [s]
margin distance
distance [cm]
margin time
time [s]
movement time
time [s]
center distance
distance [cm]
center time
time [s]
total rest time
time [s]
stereotypy counts
counts
number of stereotypy
activity [a.u.]
stereotypy time
time [s]

Figure S2. Locomotor performance in open-field tests, and in accelerating rotarod tests, as well as weight comparison between WT (white bars) and *Atxn2l*$^{+/-}$ (grey bars) mice at different ages, for 3 months, 6 months, 9 months, and 12 months. The diagrams show mean and s.e.m., the statistical evaluation was done with one-way ANOVA and revealed no significant changes by ATXN2L deficiency. Open field parameters quantified include horizontal motor activity, horizontal movement number, total distance travelled, vertical motor activity, vertical movement number, vertical movement time, distance traveled at margin or in corners of cage, time spent within 1 cm margin of cage walls, total time spent in movement, distance covered in the cage center, time spent in the cage center, total time spent in rest, count of beam breaks during a period of stereotypic activity, number of stereotypic activities, and total amount of time in stereotypic behavior. n=12vs12.

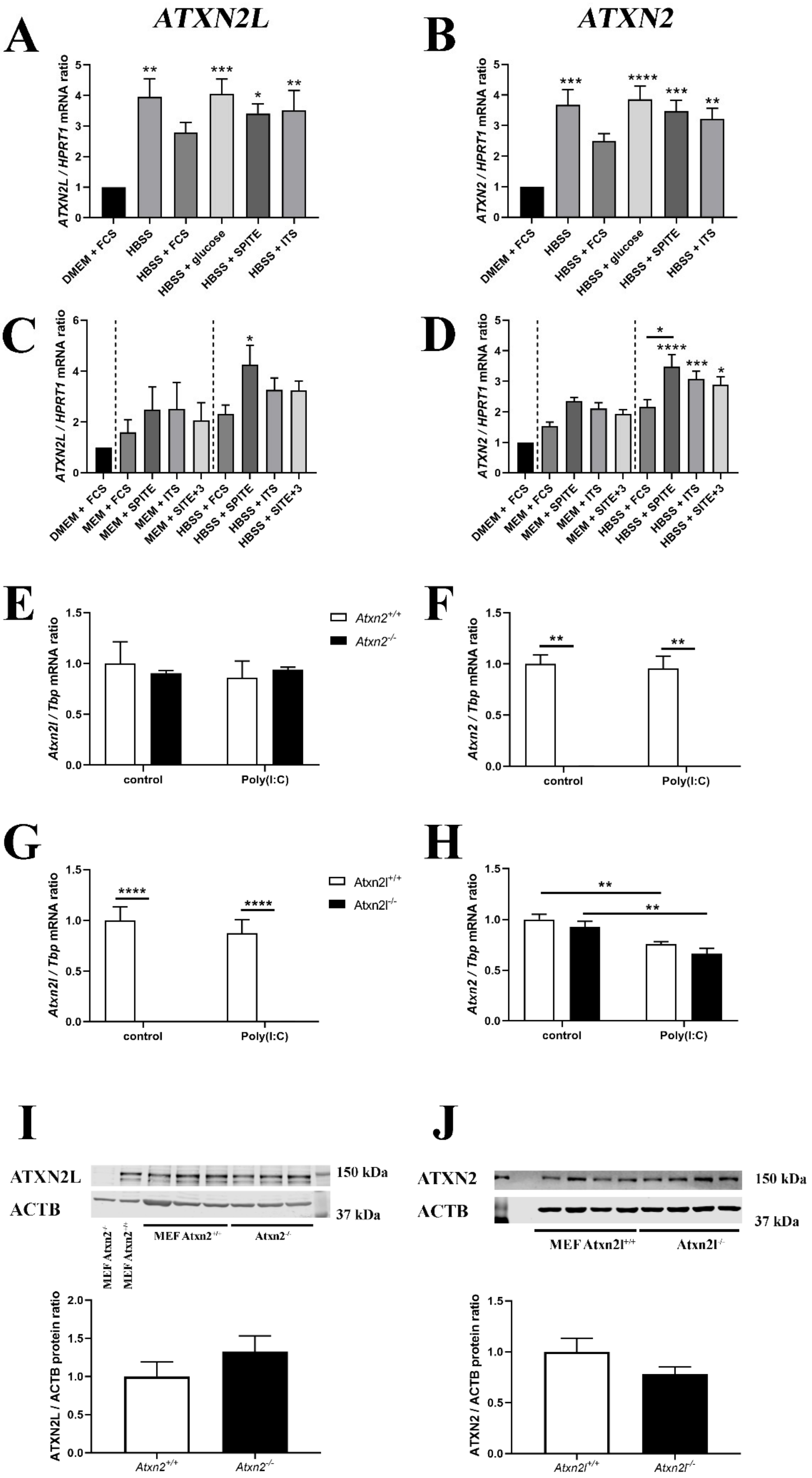
A
ATXN2L
ATXN2L / HPRT1 mRNA ratio
**
***
*
**
DMEM + FCS
HBSS
HBSS + FCS
HBSS + glucose
HBSS + SPITE
HBSS + ITS
B
ATXN2
ATXN2 / HPRT1 mRNA ratio
***
****
***
**
C
ATXN2L / HPRT1 mRNA ratio
MEM + FCS
MEM + SPITE
MEM + ITS
MEM + SITE+3
HBSS + SITE+3
D
ATXN2 / HPRT1 mRNA ratio
E
Atxn2l / Tbp mRNA ratio
Atxn2+/+
Atxn2-/-
control
Poly(I:C)
F
Atxn2 / Tbp mRNA ratio
G
Atxn2l / Tbp mRNA ratio
****
Atxn2l+/+
Atxn2l-/-
H
Atxn2 / Tbp mRNA ratio
I
ATXN2L
ACTB
150 kDa
37 kDa
MEF Atxn2-/-
MEF Atxn2+/+
MEF Atxn2+/+
Atxn2-/-
ATXN2L / ACTB protein ratio
J
ATXN2
ACTB
150 kDa
37 kDa
MEF Atxn2l+/+
Atxn2l-/-
ATXN2 / ACTB protein ratio
Atxn2l+/+
Atxn2l-/-

Figure S3. (A, B) The mRNA expressions of *ATXN2L* and *ATXN2* in human SH-SY5Y neuroblastoma cells were analyzed after incubation with starvation medium HBSS, again supplemented with 10% FCS and glucose, and now additionally with SPITE and ITS to test which component of FCS is crucial for the rescue of *ATXN2L* induction (n=5). **(C/D)** Incubation in MEM medium and HBSS in comparison, supplemented with FCS, SPITE, ITS and SPITE+3. MEM as basal medium contains more nutrients than HBSS and resulted in less strong inductions (n with MEM=3, n with HBSS=5). **(E/F)** *Atxn2l* and *Atxn2* mRNA expression in MEF with Knock-Out of *Atxn2* after incubation with the double-stranded RNA-analogue Poly(I:C). *Atxn2l* expression was unchanged in the absence of *Atxn2*, as well as after incubation with Poly(I:C). *Atxn2* mRNA was not detectable in Knock-Out MEF (n= 3 WT vs 3 $Atxn2^{-/-}$ lines). **(G/H):** *Atxn2l* and *Atxn2* mRNA expression in MEF with Knock-Out of *Atxn2l* after incubation with Poly(I:C). *Atxn2l* expression was not detected in mutant MEF and *Atxn2* responded slightly upon exposure (n = 4 WT vs. 4 $Atxn2l^{-/-}$ lines). **(I/J)** ATXN2 protein levels in $Atxn2l^{-/-}$ MEF (n=4) were unchanged, as well as ATXN2L protein levels in $Atxn2^{-/-}$ MEF (n=3) versus littermate wildtype controls. Bar diagrams below visualize the quantification, variance, and lack of significance. Statistical testing was done by unpaired t-test with Welch's correction, one-way or two-way ANOVA, significance levels were illustrated by asterisks: * $p < 0.05$; ** $p < 0.01$; *** $p < 0.001$; **** $p < 0.0001$. Comparison was always done versus nutrient abundance control condition if not stated otherwise.
